# Simulation and Optimization of SNAP-Taper Coupling System in Displacement Sensing

**DOI:** 10.3390/s21092947

**Published:** 2021-04-22

**Authors:** Jian Chen, Yongchao Dong, Han Wang, Penghui Sun, Xueliang Zeng

**Affiliations:** State Key Laboratory of Precision Electronic Manufacturing Technology and Equipment, Guangdong Provincial Key Laboratory of Micro-Nano Manufacturing Technology and Equipment, Mechanical and Electrical Engineering, Guangdong University of Technology, Guangzhou 510006, China; cnjian@mail2.gdut.edu.cn (J.C.); wanghangood@gdut.edu.cn (H.W.); 2111901091@mail2.gdut.edu.cn (P.S.); 2112001125@mail2.gdut.edu.cn (X.Z.)

**Keywords:** SNAP, displacement sensing, resonance spectrum, transmittance

## Abstract

Sensing applications based on whispering gallery mode (WGM) microcavities have attracted extensive attention recently, especially in displacement sensing applications. However, the traditional displacement sensing scheme based on shift in a single resonance wavelength, has a lot of drawbacks. Herein, a novel displacement sensing scheme based on the surface nanoscale axial photonics (SNAP) is proposed to achieve a wide range and high-resolution displacement sensor through analyzing the transmittance of multiple axial modes. By analyzing the surface plot of the resonance spectrum with different coupling positions, the ideal coupling parameters and ERV for displacement sensing are obtained. In the following, displacement sensing with high sensitivity and a wide range is theoretically realized through adjusting the sensitivity threshold and the number of modes. Finally, we present our views on the current challenges and the future development of the displacement sensing based on an SNAP resonator. We believe that a comprehensive understanding on this sensing scheme would significantly contribute to the advancement of the SNAP resonator for a broad range of applications.

## 1. Introduction

Due to the advantages of high-quality factor and low mode volume, the whispering gallery mode (WGM) microresonators have great potential for growth in many research and technology fields. In particular, WGM resonators have been extensively investigated and used in sensor fields over the last decade [1,2,3], such as biochemical substances detection [4,5], refractive index sensing [6,7], pressure monitoring [8,9], and displacement sensing [10,11,12,13,14]. In the study of displacement sensing, two conventional mechanisms have attracted widespread interest. The first conventional method is tracing the resonance wavelength shift caused by the deformation of a resonator. For instance, V.S. Ilchenko et al. demonstrated the tunability of WGM in a SiO${}_{2}$ microsphere resonator by applying mechanical strain, indicating the potential for a displacement sensing application with a theoretical resolution of better than 0.1 nm [15]. Similarly, M. Sumetsky et al. realized tensile tuning with a wide range and small driving force based on the SiO${}_{2}$ resonators, and a theoretical resolution of the displacement sensing better than 0.1 nm [16]. The second method is monitoring the resonance wavelength shift, resulting from the change in coupling conditions. In this regard, Song Yuejiang et al. obtained a wavelength tuning slope of 95.86 pm/nm by moving the tapered microcylinder resonator along its axis, as well as the resolution of 0.29 nm [17].

Although the sensors with superior sensitivity can be obtained according to the above two schemes, sensing based on the shift in a single resonant wavelength has low accuracy, because of undesired factors such as external temperature change. In previous approaches, sensing was achieved by monitoring the relative shift in a resonant mode induced by a displacement change with respect to its original state. Therefore, on the one hand, it is crucial to fundamentally eliminate the surrounding temperature noise to realize the high-accuracy displacement sensing; on the other hand, it is impossible to determine the absolute value of the displacement only from the WGM spectrum without knowing the initial displacement. The surface nanoscale axial photonics (SNAP) microresonator proposed by M.Sumetsky has a stable, regular resonance spectrum and a long WGM field distribution in the axial direction [18], indicating the potential application of a wide-range and high-sensitivity displacement sensing. We have investigated the variation characteristics of the resonance spectrum brought by the displacement of a SNAP microresonator [19,20], but we only focused on the property change in a single axial mode, leading to the displacement sensing with a narrow range and low accuracy.

To solve this problem, one ingenious method, using the variation characteristics of multiple axial modes in a SNAP resonator, is proposed in this paper. The position information is represented by different resonance spectrum characteristics. Through the reasonable application of multiple axial modes, the unique resonance spectrum characteristics can be obtained at any position. At the same time, the absolute position of the resonator can be determined by the combination of multiple modes. Therefore, we could derive the actual displacement from the overall pattern of the spectrum. It is worth noting that this measurement method is not affected by temperature and does not require initial displacement. Resonance spectra were obtained through theoretically simulating a SNAP coupling system by MATLAB, and the effect of coupling parameters and effective radius variation (ERV) on the transmission spectra are investigated. The working principle of the displacement sensor based on a SNAP-taper coupling system is presented in Section 2. In Section 3, the theory of WGM in SNAP and the theory of SNAP-taper coupling system are introduced, which are based on the theoretical model of SNAP resonator proposed by M. Sumetsky [21]. In Section 4, simulation results are presented and the coupling parameters, as well as the ERV of the SNAP-taper system, are optimized. Then, based on the resonant spectrum of the determined parameters, the principle and feasibility of using multi-order modes for sensing are explained in detail. At last, some meaningful results a discussion of follow-up work are summarized in Section 5.

## 2. Sensor Concept

**Figure 1:**
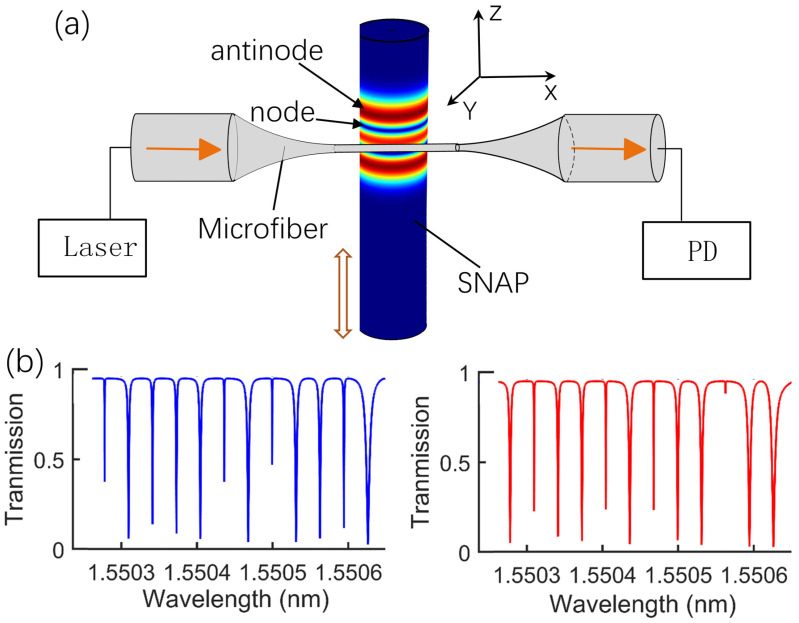
SNAP displacement detection principle. (**a**) Schematic illustration of SNAP-based displacement sensing device and operation principle. The colored plotting describes the simulated spatial distribution of high-Q local modes. (**b**) Two different normalized transmission spectra in the sensing process.

Figure 1a shows the displacement sensing schematic of the SNAP-taper coupling system. The SNAP microresonator is positioned to be in contact with and perpendicular to the tapered fiber. The displacement is introduced by the SNAP moving along the z-axis. The incident laser is coupled into the resonator via a tapered fiber, which is used to excite the WGMs in the resonator. Different localized WGMs are excited when the fiber taper couples with the SNAP at a different position. As we know, microresonators with equidistant spectra over tens of axial modes have been demonstrated in SNAP [22,23]. Meanwhile, electromagnetic field intensity distributions are unequal in different axial modes and various coupling positions, which means that spectra with different characteristics can be obtained when the SNAP position relative to the taper changes, as shown in Figure 1b. Thus, the transmission spectrum characteristics can be utilized to measure displacement. Compared with the conventional displacement-sensing principle based on strain-induced shape changes, the effectiveness and stability of the proposed method will be proven in this paper.

Figure 1b shows the normalized transmission spectra with the tapered fiber positioned in two different regions of the SNAP. These resonant dips represent different orders of axial modes supported in SNAP, respectively. As the transmission spectrum with the equidistant free spectral range (FSR) was demonstrated in the SNAP with parabolic ERV, the SNAP with parabolic is chosen in the following investigation [24,25,26,27]. Additionally, the coupling efficiency between the tapered fiber and the SNAP resonator is determined by the overlapping integral of their evanescent fields. Therefore, the resonance intensity is varied for different-order axial modes, which is significantly manifested by the width and depth of resonance dips. Similarly, for the same mode, the resonance dips are also different when the coupling fiber is placed in different positions of the SNAP. Based on this characteristic, the SNAP mode can be utilized for displacement sensing. However, with respect to a single mode, the distance between the adjacent antinode and node (marked in Figure 1a) is so narrow that a large range of displacement sensing cannot be achieved. With respect to the above factors, we propose a novel displacement sensing scheme comprehensively using the multi-order axial modes of SNAP.

For displacement sensing based on the SNAP resonator, sensitivity, accuracy, and detection range are three important parameters to describe the performance. The sensitivity has a lot to do with the changing amplitude of transmittance, or a Q factor resulting from the unit displacement variation. The accuracy is determined by the anti-noise capability. Compared with the traditional scheme, the proposed method can get rid of the interference of irrelevant external factors, like temperature variation. The detection range depends on the distribution status of the WGM field along the axial direction. A larger sensing range is attributed to the wider axial distribution. Consequently, in order to improve the sensing performance, it is necessary to investigate the effects of coupling parameters and ERV profile on the resonance spectrum.

## 3. Theory

### 3.1. WGM in SNAP

Due to its highly prolate shape, the SNAP has a class of WGMs with advantageous properties. Additionally, the performance of SNAP devices depends only on the ERV of the fiber, which combines the contributions of nanoscale variations of the fiber physical radius and its refractive index. According to the article [28], the parabolic radius profile of the SNAP resonator is expressed by

(1) $$R\left(z\right)=R_{\left(0\right)}\cdot \left(1-1/2{\left(\Delta k\cdot z\right)}^{2}\right)$$

where Δk denotes the curvature of the resonator profile, $R_{\left(0\right)}$ is the maximum radius of the resonator at z = 0. As discussed in references [28,29], the axial wave equation, which only depends on the z coordinate, is represented as

(2) $$\left(\partial_{z}^{2}+k_{z}^{2}\right)\cdot \Psi \left(z\right)=0$$

where $k_{z}$ is the axial component of wave vector. Then, according to the derivation of Maxwell’s equations to solve the radial wave equation and axial wave equation, the normalized axial field distribution function of the parabolic SNAP is given as $I_{norm}=\Psi^{2}/max\left(\Psi^{2}\right)$, where Ψ(z) is the eigenfunction for the axial wave equation, describing the field distribution in axial direction.

### 3.2. Theory of a SNAP-Taper Coupling System

To gain the transmission spectrum of the SNAP coupling system, the theoretical model based on the one-dimensional Schrödinger equation was constructed to describe multiple resonances. The transmission spectrum of the SNAP coupling system is given by Sumetsky [18,21]

(3) $$S\left(\lambda \right)=S^{\left(0\right)}-\frac{i\Lambda_{n}}{\left(E\left(\lambda \right)-E_{n}\right)-\Delta_{n}+i\left(\Gamma_{0}+\Sigma_{n}\right)}$$

(4) $$\Lambda_{n}={\left|C\right|}^{2}\Psi_{n}^{2}\left(z\right),\Delta_{n}=-Re\left(D\right)\Psi_{n}^{2}\left(z\right),\Sigma_{n}=Im\left(D\right)\Psi_{n}^{2}\left(z\right)$$

where λ and z are independent variables, representing the wavelength and the axial coupling position, respectively. *C* and *D* are coupling parameters. $S^{\left(0\right)}$ is the radiation loss parameter, which indicates the non-resonant component of the transmission amplitude S(λ). E(λ) is the energy proportional to the variation in resonant wavelength. $E_{n}$ corresponds to the energy eigenvalue of the *n*-th order axial mode. $\Psi_{n}\left(z\right)$ is the axial field distribution function of the *n*-th order axial mode. $\Gamma_{0}=\left(8\pi^{2}n_{f0}^{2}/\lambda_{res}^{2}\right)\gamma_{res}$ is the linewidth of the resonance due to the propagation losses in SNAP. $n_{f0}$, $\lambda_{res}$ and $\gamma_{res}$ stand for the refractive index of fiber, resonant wavelength, and attenuation parameter, respectively.

Considering the loss in the actual coupling process, the energy circulating in the microresonator cannot be infinite. From the law of conservation of energy, the parameters $S^{\left(0\right)}$, ${\left|C\right|}^{2}$ and D must be restricted by the inequalities

(5) $$|S^{\left(0\right)}|<1$$

(6) $$Im\left(D\right)>{\left|C\right|}^{2}\frac{1-Re(S^{\left(0\right)})}{1-{\left|S^{\left(0\right)}\right|}^{2}}$$

Next, we conducted a parametric study of the transmission spectrum. Apparently, changing so many parameters simultaneously is not suitable for accurate analysis of the resonance spectrum. Therefore, we only changed one parameter at a time.

### 3.3. Simulation Parameters

In order to calculate the optical field distribution of the SNAP resonator, and determine the resonance spectrum of the SNAP-taper coupling system for displacement sensing, numerical calculations of the coupled system using MATLAB are carried out. The simulation parameters are $n_{f0}=1.452$, $\gamma_{res}=0.05$ pm, $R_{\left(0\right)}=62.4567$ µm. According to the above, the coupling parameters and ERV have a great impact on the resonance spectrum characteristic. Initially, we set coupling parameters ${\left|C\right|}^{2}=0.015$ µm${}^{-1}$, D=0.02+0.016i µm${}^{-1}$, $S^{\left(0\right)}=0.95-0.01i$, and Δk=0.012 µm${}^{-1}$. The angular mode number is initially set to m = 360, and the corresponding resonance wavelength is $\lambda_{res}=1.5507$ µm.

## 4. Results and Discussion

### 4.1. Influence of Coupling Parameters and ERV on the Resonance Spectrum

As we know, in a conventional microresonator-taper coupling system, a sufficient overlap between the evanescent fields is a key requirement for realizing efficient coupling. Unlike spheroidal microresonator, SNAP supports localized axial WGM modes and delocalized radiation modes, their interaction is similar to the energy level of the outer layer of an atom. In different coupling situations, the interference between the two parts in the coupled system will produce different peak shapes [30]. Furthermore, the lineshape has a great impact on the sensor performance. Therefore, we focus on the variation in lineshape by first optimizing the parameters. As discussed in reference [30], parameter $S^{\left(0\right)}$ is corresponding to the change in taper waist diameter: when the taper waist diameter decreases, lineshape from Lorentzian dip to electromagnetically induced transparency (EIT) peak is obtained. Therefore, we first study the effect of $S^{\left(0\right)}$ on the spectrum to adjust the lineshape of resonant dips.

**Figure 2:**
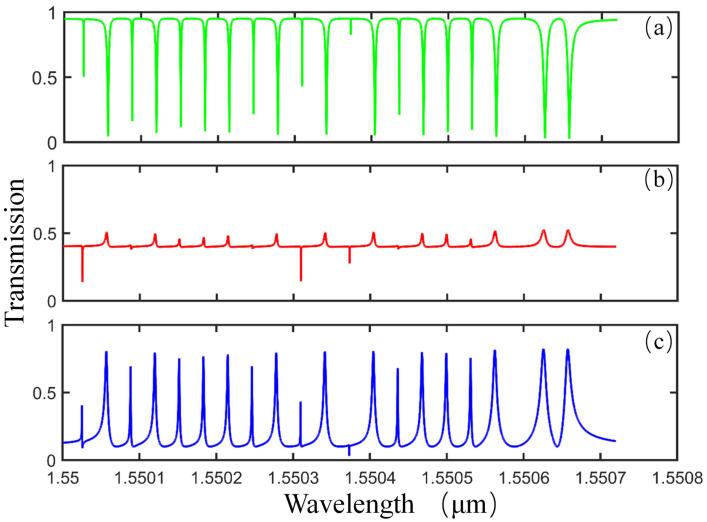
The normalized transmission spectrum of SNAP-taper coupling with different $S^{\left(0\right)}$. (**a**) $S^{\left(0\right)}=0.95-0.01i$; (**b**) $S^{\left(0\right)}=0.40-0.01i$; (**c**) $S^{\left(0\right)}=0.10-0.01i$.

The normalized transmission spectra under different parameter $S^{\left(0\right)}$ are plotted in Figure 2. From Figure 2a–c, the Lorentzian dip, Fano peak, and EIT peak are shown respectively, with ${\left|C\right|}^{2}=0.015$ µm${}^{-1}$, D=0.02+0.016i µm${}^{-1}$ and different $S^{\left(0\right)}$ shown in the caption. As we know, in practical applications, especially in the field of sensing, a stable and regular resonance spectrum is essential [31]. As shown in Figure 2a, the lineshape of transmission spectrum is shown as multiple symmetric and equidistant Lorentzian dips with low transmittance, which makes the spectrum an ideal tool for realizing displacement sensing due to the superiority in accurate recognition and wavelength tracking of the resonant modes. Along with the real part of radiation loss parameter $R_{e}\left(S^{\left(0\right)}\right)$ decreasing to 0.40, the lineshape of WGMs changes to the Fano resonance, as shown in Figure 2b. When $R_{e}\left(S^{\left(0\right)}\right)$ continues to decrease, the lineshape of transmission coverts into an EIT peak with $R_{e}\left(S^{\left(0\right)}\right)=0.10$ in Figure 2c. Compared with the Lorentzian dips, which have high Q factors and low transmittance, the stability deterioration of Fano and EIT dips leads to a bad performance in displacement sensing. In order to guarantee the sensing performance, we choose $S^{\left(0\right)}$ with a real part equal to 0.95 in the following calculations.

**Figure 3:**
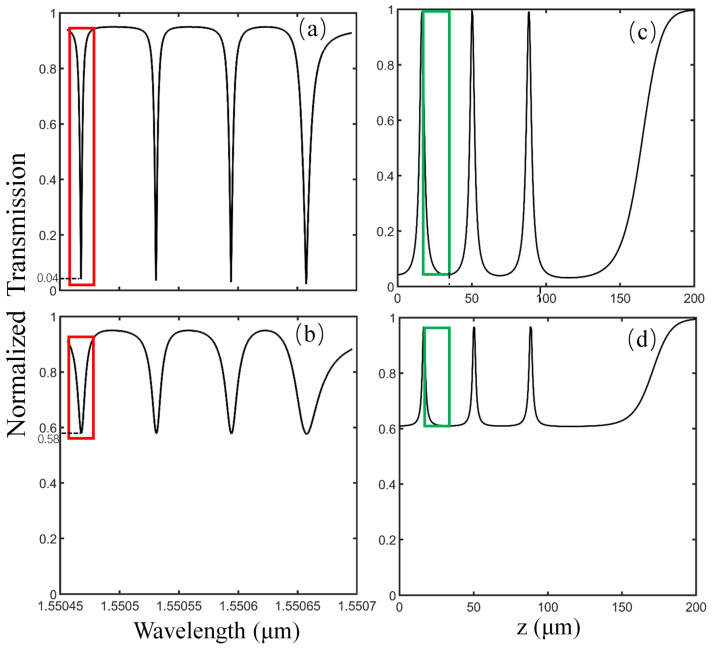
(**a**,**b**) Normalized transmission spectrum with different coupling parameter D equal to 0.016i µm${}^{-1}$, 0.04i µm${}^{-1}$, respectively. (**c**,**d**) The normalized transmittance-z curve when q = 6 corresponding to (**a**,**b**).

Next, to gain more insight into the effect of the coupling parameter on the transmittance mentioned above, calculations are performed with different parameter D. The effect of the imaginary part of D (Im(D)) on the resonant modes is studied first. With Im(D) changing from 0 to 0.2, the simulation results indicate that Im(D) only describes the shift in the resonant wavelength, which is the same as the discussion in reference [32]. Since the phase shift has no effect on the Q factor and transmittance of resonant modes, for simplicity’s sake, $R_{e}\left(D\right)$ is set to be zero when we investigate the imaginary part of D (Im(D)). Figure 3a,b show the normalized transmission spectra of the fourth- to seventh-order axial modes with varied parameters Im(D) equal to 016*i* and 0.04*i*, respectively. It can be seen that the transmittance (0.04) in Figure 3a is much lower than that (0.58) in Figure 3b. As we know, lower transmittance means less coupling loss, giving rise to a higher sensitivity in the sensing process. Therefore, we choose Im(D)=0.016i µm${}^{-1}$ in the following calculations. Figure 3c,d are the transmittance-z curves of the seventh-order axial mode, corresponding to the mode in the rectangular selection in Figure 3a,b, respectively. Note that the slope in the transmittance-z curve represents the sensitivity of our proposed displacement sensor. It is obvious that the slope of curve in the green rectangular selection in Figure 3c is monotonous and is larger than that in Figure 3d, which is beneficial to achieving a higher displacement sensing resolution. Consequently, in order to obtain high sensitivity in displacement sensing, the adjustment of Im(D) is essential.

**Figure 4:**
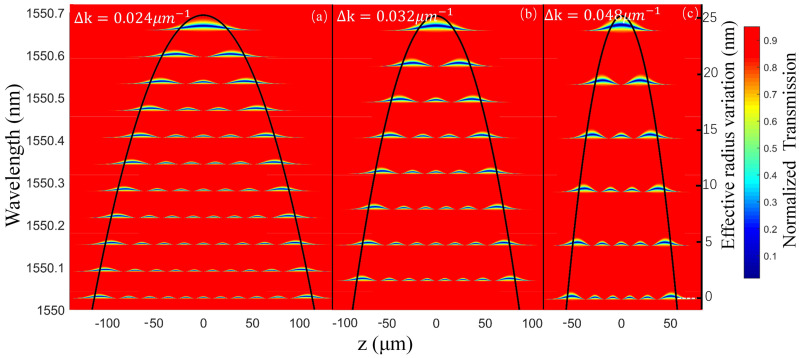
The surface plot of the resonance transmission amplitude of the SNAP microresonator with different curvatures, and the black curve is the envelope of the resonance spectrum. From (**a**) to (**c**), Δk = 0.024 µm${}^{-1}$, 0.032 µm${}^{-1}$, 0.048 µm${}^{-1}$, respectively. The coupling parameters are ${\left|C\right|}^{2}$ = 0.015 µm${}^{-1}$, D = 0.02 + 0.016i µm${}^{-1}$, $S^{\left(0\right)}=0.95$ - 0.01i.

Besides the coupling parameters, the curvature Δk of the parabolic SNAP resonator profile also has a strong influence on the spectrum characteristics. To find the effect of Δk on the resonant spectrum, the axial intensity distribution function of the modes is calculated and the coupling property is deeply investigated with different curvatures. With the coupling parameters ${\left|C\right|}^{2}=0.015$ µm${}^{-1}$, D=0.02+0.016i µm${}^{-1}$ and $S^{\left(0\right)}=0.95-0.01i$, the results of the calculation when Δk is set to be 0.024 µm${}^{-1}$, 0.032 µm${}^{-1}$ and 0.048 µm${}^{-1}$, respectively, are presented by the surface plot in Figure 4, which depicts the transmission amplitude as a function of the axial position of the SNAP resonator and wavelength. This surface plot quantitatively reproduces the position of the nodes and antinodes of the SNAP mode, and the envelop of the spectral resonances shows close agreement with the parabolic ERV of SNAP profile, which coincides with the rescaling relation between the wavelength variation and the ERV [32]. In addition, it should be pointed out that, as the Δk increases from 0.024 µm${}^{-1}$ to 0.048 µm${}^{-1}$, the wavelength between adjacent axial modes gradually augments. As we know, a large FSR can effectively avoid mode overlap, thus reducing the error rate of mode recognition, which is beneficial, with practical applications in sensing. Despite this, there are two disadvantages for a too-large FSR with respect to our proposed displacement sensing method here. Firstly, scanning a fixed number of modes requires a wider frequency-scanning range, which is a huge test for the performance of the instrument when a high wavelength resolution is necessary. As shown in Figure 4, the number of modes contained in the same wavelength range is different, with eleven modes for 0.024 µm${}^{-1}$ and only six modes for Δk=0.048 µm${}^{-1}$, respectively. Secondly, the fabrication of the SNAP resonator with a large FSR and enough high-Q axial modes, which depends on the ERV height limited by the fiber property, is challenging due to the state-of-the-art technique. Therefore, there should be a tradeoff between the FSR and the present fabrication technique. In this case, we choose Δk=0.012 µm${}^{-1}$ in the following analysis for displacement sensing.

### 4.2. Displacement Sensing Analysis

**Figure 5:**
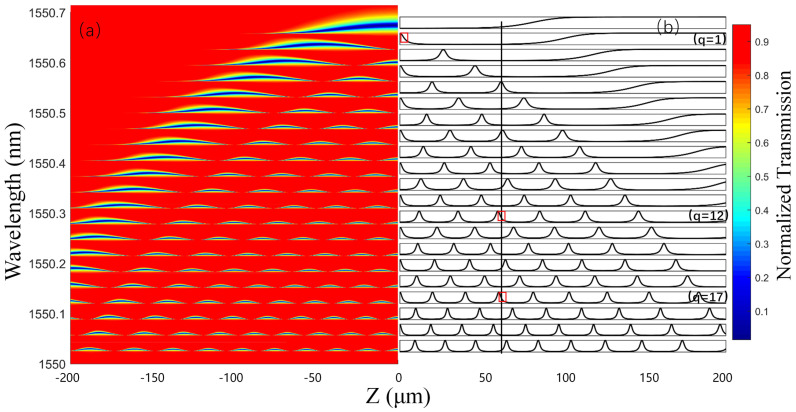
(**a**) The ideal surface plot of the resonance transmission amplitudes of the SNAP, with the coupling position from z = −200 µm to z = 0 µm. (**b**) The transmittance-z curves of the 21 lowest-order axial modes (q = 0 20), with the coupling position from z = 0 µm to z = 200 µm.

According to the above research, the influence of coupling parameters and ERV on the transmission spectrum is acquired by the MATLAB simulations. Obviously, the resonance spectrum characteristics are not only related to a single parameter but affected by the combination of multiple parameters, and there will be checks and balances between these parameters. The normalized transmission amplitudes surface plot with relatively balanced characteristics is given in Figure 5a, where simulation parameters are Δk=0.012 µm${}^{-1}$, ${\left|C\right|}^{2}=0.015$ µm${}^{-1}$, D=0.02+0.016i µm${}^{-1}$, $S^{\left(0\right)}=0.95-0.01i$. Considering the symmetry of the transmission amplitudes at about the center of SNAP (z = 0), only a half of the surface plot is shown, while the other half is displayed as curves in the relationship between the transmittance and z for different axial modes.

Figure 5b shows the transmittance-z curves of the 21 lowest-order axial modes (q = 0∼20) with the same simulation parameters as Figure 5a. For the q = 1 axial mode, as the coupling position changes from z = 200 µm to z = 0 µm, the coupling strength experiences the undercoupling regime through the critical coupling point, overcoupling regime and, at last, back to the undercoupling regime. Accordingly, the transmittance-z curve experiences a process consisting of flatness, decline, flatness and incline, completing what we call a whole cycle. For q = 0 axial mode, the transmittance-z curve goes through a half cycle. Note that, compared with the q = 0 mode, the transmittance-z curve of q = 1 mode has a both monotonous and steep region (marked by a red box in Figure 5b) near the SNAP center, which can be applied to realize high-resolution displacement sensing. The high steep curve region is determined by the position of nodes in the axial mode field intensity distribution. Similarly, for the q = n axial mode, the transmittance-z curve experiences (n + 1)/2 cycle, leading to n regions that are monotonous and steep. Consequently, by increasing the number of supported axial modes in a SNAP resonator, more monotonous and steep regions available for displacement sensing can be obtained, so that the sensing range will augment. In addition, for the higher-order axial modes, the length of the steep regions decreases, giving rise to a better performance of the sensing resolution. To further illustrate the displacement sensing principle, as shown in Figure 5b, a position indicated by a vertical line is randomly selected and considered, where q = 12 and 17 modes (selected by red boxes) work on the condition of undercoupling and subsequently exhibit a steep curve. As a result, the randomly selected position possesses the ability to realize the displacement sensing with high sensitivity (though a small sensing range). Besides q = 12 and 17 modes for the considered position, there are a host of other modes that can be utilized to determine the absolute position of the SNAP resonator. By combining the transmittance of multiple axial modes, a larger sensing range with high resolution can be achieved.

**Figure 6:**
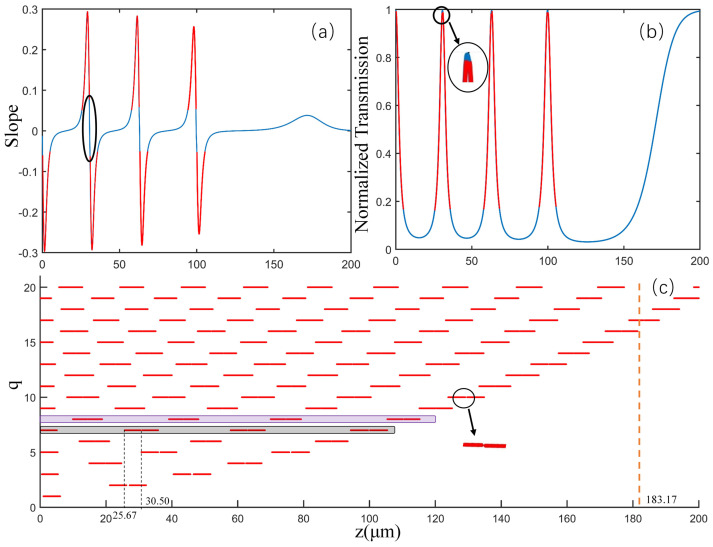
More detailed descriptions of the sensing methods. (**a**) Slope of the transmittance-z curve for q = 7 mode. (**b**) The transmittance -z curve of q = 7 mode. (**c**) Range available for sensing in q = 0~20 mode.

To deeply investigate the sensitivity and measurement range of the proposed displacement sensor, we calculate the derivative of the transmittance-z curve with respect to z for all the axial modes and the obtained slope of the transmittance-z curve for q = 7 mode is given as an example in Figure 6a. It can be observed that the slope curve possesses three acute peaks and four dips which, corresponding to seven steep regions of the transmittance-z curve in Figure 6b, allows for a high sensitivity in displacement sensing. Obviously, a large absolute value of slope represents a high sensitivity and, as a result, we should choose the region with a large absolute value of slope to realize high-sensitivity displacement sensing. However, as the required sensitivity increases, the obtainable sensing range will significantly decrease, limiting the sensing performance. Therefore, there should be a tradeoff between the sensitivity and measurement range. Considering this, the absolute value of slope is set to be 0.05 as a threshold. By comparing this with the threshold level, the sensing regions with high sensitivity are obtained and plotted using red lines in Figure 6a,b. Note that the region selected by an ellipse box is located at the node of WGM field, where the coupling strength is nearly zero, thereby inducing a turning point of the transmittance-z curve and discretized high-sensitivity regions.

Figure 6c displays the discretized high-sensitivity regions for the 21 lowest-order axial modes. It can be found that the number of the high sensitivity regions for q = n axial modes is n and these regions of different-order modes are staggered, which offers the potential for a SNAP resonator to realize the continuous displacement measurements with a wide range and high sensitivity. For example, the separated regions for q = 7 mode are: 0.25∼5.08 µm, 25.67∼30.50 µm, 31.00∼35.83 µm, 57.83∼62.75 µm, 63.25∼68.25 µm, 94.33∼99.58 µm and 100.2∼105.4 µm, respectively, while q = 8 mode corresponds to: 9.92∼14.25 µm, 15.50∼18.83 µm, 39.08∼43.42 µm, 43.47∼28.08 µm, 70.00∼74.42 µm, 74.83∼79.25 µm, 105.42∼110.166 µm, 110.58∼115.25 µm. These regions are spatially staggered and complementary, allowing for an increased measurable regions with high sensitivity. Though not given here, using the transmittance of all the q = 0∼20 axial modes, a displacement sensor with a measurement range of 183.17 m and sensitivity (described by the transmittance variation versus displacement) more than 0.05/µm is numerically demonstrated. If the minimum distinguishable transmittance change is 1/1000, the obtainable displacement resolution will be 20 nm. Apparently, the displacement resolution depends on the slope threshold (shown in Figure 6a). Thus, by increasing the number of axial modes and the slope threshold, the resolution can be further improved.

The simulation results above theoretically demonstrate the effectiveness and feasibility of the proposed SNAP-taper coupling system for displacement sensing. It can be found that the sensing mechanism is based on the transmittance of the multiple axial modes and thus exhibits a good temperature stability. Benefiting from enough axial modes and optimized coupling parameters of the SNAP resonator, a wide range and high-resolution displacement sensor can be achieved. However, in order to realize this in experiments, there are some obstacles that need to be solved. First, it is obvious that the sensing performance is highly affected by the SNAP’s ERV, which determines the number of excited axial mode. Usually, the SNAP resonator is fabricated by accurately modifying the optical fiber-effective radius at nanoscale based on CO${}_{2}$ laser or UV (248 nm excimer laser) beam exposures; as a result, the ERV is limited by the fiber inherent property and a new method to fabricate the SNAP with a large ERV is in urgent demand. Second, data-processing, including spectrum sampling, mode recognition and the calculation of mode characteristic parameters, is significantly important in realizing high-resolution displacement sensing. Based on the calculated characteristic parameters of different axial modes, the SNAP displacement can be obtained.

Notably, the fiber-based SNAP resonator can be fabricated into a probe-type sensor, making it a powerful tool for many displacement sensing applications, such as microstructure measurements, in both aerospace and nano-lithography fields. In addition, an algorithm to directly and efficiently determine the absolute position of the SNAP from the WGM spectrum is urgently needed. Further work will be focused on the experiment realization and the development of this algorithm, such as an encoding method or a neural network model.

## 5. Conclusions

In conclusion, we propose a novel displacement sensing method based on the multi-order modes of the SNAP-taper system. The sensing principle is no longer based on the shift in a single resonant wavelength, but utilizing the transmittance of the axial modes. The resonance spectrum and transmittance-z curve of SNAP-taper system are obtained by MATLAB simulation. By analyzing the effect of coupling parameters and ERV on the performance of displacement sensing, we obtain the best spectral shape when ${\left|C\right|}^{2}=0.015$ µm${}^{-1}$, D=0.02+0.016i µm${}^{-1}$, $S^{\left(0\right)}=0.95-0.01i$, Δk=0.012 µm${}^{-1}$. Apart from this, in order to further illustrate the sensing principle, we investigate the effect of the single-order, multi-order, and threshold on the sensing performance. This is feasible to realize wide-range and high-sensitivity displacement sensing by adjusting the transmittance threshold and the number of modes. Additionally, in the case of the 21 lowest-order modes, the sensing range can reach 183.17 µm with a resolution of 20 nm, when the sensitivity threshold is set at 0.05 and a distinguishable transmittance change of 1/1000 is considered. Although this new sensing scheme is theoretically feasible, there are still huge challenges in the experiment realization, such as the precise manufacturing of regular SNAP shapes. Future studies will explore this sensing issue by establishing experimental platforms. In addition, future research might apply neural networks to process a large amount of data in the transmission spectrum to realize the displacement sensing.

## Data Availability

Not applicable.
